# Effects of different types of radiation therapy on cardiac-specific death in patients with thyroid malignancy

**DOI:** 10.3389/fcvm.2022.996732

**Published:** 2022-11-10

**Authors:** Ruxin Wang, Haowen Ye, Ying Wang, Li Ma, Jinjing Wei, Xiaofang Zhang, Lihong Wang

**Affiliations:** ^1^Department of Endocrinology and Metabolism, The First Affiliated Hospital of Jinan University, Guangzhou, China; ^2^Department of Functional Examination, Gansu Provincial Maternal and Child Health Hospital, Lanzhou, China; ^3^Clinical Experimental Center, The First Affiliated Hospital of Jinan University, Guangzhou, China

**Keywords:** radiation therapy, heart, thyroid malignant tumors, death of heart disease, cardiac death

## Abstract

Radiation therapy (RT) is one of the common and widely used treatment method for thyroid tumors. Considering that the thyroid is located close to the heart, the radiation generated during the treatment of thyroid tumors may have an adverse greater impact on the heart. This study is to explore the influencing factors, especially additional effects of RT, on cardiac-specific death among patients with malignant thyroid tumors. Collecting information from the National Cancer Institute’s Surveillance, Epidemiology, and End Results (SEER) database using SEER*Stat. Patients with malignant thyroid tumors were searched, whether receiving RT or not. Ultimately, 201, 346 eligible patients were included. Propensity Score Matching (PSM) was used to minimize bias of baseline characteristics by adjusting for confounding factors. COX (proportional hazards) and fine-gray (competing risk) model regression analysis were used to explore the effects of various influencing factors on cardiac-specific death. The present analysis showed that, compared with non-RT, RT based upon radioactive implants and beam radiation were associated with lower risk of cardiac-specific death in patients with thyroid malignancy, beam radiation therapy may had a similar effect. Besides, the remaining RT methods did not significantly increase the risk of cardiac-specific death. In addition, Asian or Pacific Islander ethnicity, female sex, marital status, combined summary stage (localized, regional, and distant), high-income, and later year of diagnosis were associated with lower risk of cardiac-specific death. While older age of diagnosis, African ethnicity, non-Hispanic ancestry, and derived AJCC stage (IV) were risk factors for cardiac-specific death. These results help to identify the factors influencing cardiac-specific death among patients with thyroid malignancies. Furthermore, it may helps to improve the clinical application of RT without too much concern about adverse cardiac effects.

## Introduction

While radiation therapy (RT) can improve the condition of patients with cancer, it can also cause harmful off-target side, such as radiation-induced heart diseases, which presents a major concern in cancer therapy. Under the course of RT treatment – particularly for malignant tumors – the accumulation of high doses of radiation can lead to delayed-onset cardiac damage and, in some cases, cardiac death, which can occur years or even decades after radiation exposure (1). Current studies have demonstrated that RT significantly increases the risk of cardiac death in some tumors (2–4), as RT is widely used in the treatment of thyroid tumors (5), and considering that the thyroid is located near the heart, the present study sought to explore whether RT in the context of thyroid cancer treatment may be associated with increased rates of cardiac-specific death.

The National Cancer Institute’s Surveillance, Epidemiology, and End Results (SEER) database is a population-based coordinated state cancer registry that collates the demographic and clinical information of cancer patients from multiple regions of the United States. Therefore, the SEER database is a potentially useful resource for exploring the cardiac complications of RT among patients with malignant thyroid tumors (6). In this study, we explored the incidence of post-radiation cardiovascular complications in patients with malignant thyroid tumors and identify the influencing factors of cardiac-specific death by collecting data from SEER databases (7, 8).

## Patients and methods

### Patient selection

The “Incidence” package of SEER database was accessed using SEER*Stat (version 8.4.0.1), and the data of patients with malignant thyroid tumors, whether receiving RT or not, from 2000 to 2019 was retrieved. Cardiac-specific death was defined as all deaths due to heart disease. Those who were unsure whether to receive RT were excluded. Ethical approval and patient consent were not required since the SEER data is publicly available (9).

### Statistical analysis

Patients were divided into RT and non-RT groups, according to whether or not they received radiation therapy. Firstly, we calculated the differences in baseline characteristics of the two groups, and then used regression analysis to explore the factors influencing cardiac-specific death. The missing data were filled with multiple imputation. Categorical variables were expressed as frequencies and proportions, which were compared by chi-square or Fisher’s exact test. Continuous variables were expressed as mean ± SD and compared by *t*-test if also homoscedastic. Otherwise, these variables were expressed as median and compared by Mann-Whitney *U* test. After verifying that the variables meet the equal-proportional hazards assumption, COX (proportional hazards) and Fine-Gray (competing risk) model regression analysis were used to explore the factors influencing cardiac-specific death. All statistical tests were two-sided, and *P* < 0.05 was considered statistically significant. SPSS (version, 27.0; SPSS, Chicago, IL, USA) and STATA (version, 17.0; STATA, TX, USA) were used for all statistical analyses.

### Propensity score matching

Propensity score matching (PSM) has been widely used to improve comparability between groups in observational studies by adjusting for confounding factors (10). A propensity 1:1 matching analysis was performed using SPSS to minimize bias, followed by the statistical analyses as described above.

## Results

### Patient characteristics

Ultimately, 201, 346 eligible patients were included in this study. The average age of patients at diagnosis was around 50 years old, and the patients were predominantly White ethnicity, non-Hispanic ancestry, female sex, marital status, and middle-income. About 44% of patients received various types of RT, the vast majority of patients received surgery, and very few patients were treated by chemotherapy. There were significant differences in all characteristics (Table 1).

**TABLE 1:** Characteristics of patients included in non-malignant tumor patients.

Variables	Before matching			After matching		
	Radiation [*n* (%)]	No radiation [*n* (%)]	*p*	Radiation [*n* (%)]	No radiation [*n* (%)]	*p*
Number of patients (*n*)	89,238	112,108		64,834	64,834	
Age of diagnosis (years)	48.49 ± 15.785	51.51 ± 16.039	<0.001*	49.38 ± 15.611	48.81 ± 15.956	<0.001*
**Race**						
White	73,091 (81.9)	91,800 (81.9)	<0.001*	53,159 (82.0)	53,090 (81.9)	0.319
Black	4,950 (5.5)	8,111 (7.2)		3,876 (6.0)	3,809 (5.9)	
Asian or Pacific Islander	10,540 (11.8)	11,473 (10.2)		7,353 (11.3)	7,442 (11.5)	
American Indian	657 (0.7)	724 (0.6)		446 (0.7)	493 (0.8)	
**Origin**						
Hispanic	15,855 (17.8)	17,645 (15.7)	<0.001*	11,282 (17.4)	11,272 (17.4)	0.942
Non-Hispanic	73,383 (82.2)	94,463 (84.3)		53,552 (82.6)	53,562 (82.6)	
**Sex**						
Male	23,869 (26.7)	25,707 (22.9)	<0.001*	16,331 (25.2)	16,115 (24.9)	0.166
Female	65,369 (73.3)	86,401 (77.1)		48,503 (74.8)	48,719 (75.1)	
**Marital status**						
Single	19,773 (22.2)	24,000 (21.4)	<0.001*	13,613 (21.0)	14,990 (23.1)	<0.001*
Married	57,650 (64.6)	70,728 (63.1)		42,226 (65.1)	40,989 (63.2)	
DSW	11,584 (13.0)	17,062 (15.2)		8,832 (13.6)	8,663 (13.4)	
Unmarried or domestic partner	231 (0.3)	318 (0.3)		163 (0.3)	192 (0.3)	
**Year of diagnosis**						
2000–2004	15,741 (17.6)	15,996 (14.3)	<0.001*	11,723 (18.1)	12,759 (19.7)	<0.001*
2005–2009	23,560 (26.4)	23,068 (20.6)		16,814 (25.9)	16,806 (25.9)	
2010–2014	27,089 (30.4)	32,890 (29.3)		19,263 (29.7)	17,660 (27.2)	
2015–2019	22,848 (25.6)	40,154 (35.8)		17,034 (26.3)	17,609 (27.2)	
Months from diagnosis to treatment	0.771 ± 1.263	0.669 ± 1.387	<0.001*	0.720 ± 1.246	0.781 ± 1.534	<0.001*
**Derived AJCC stage group**						
I	52,577 (58.9)	82,551 (73.6)	<0.001*	42,634 (65.8)	43,672 (67.4)	<0.001*
II	7,538 (8.4)	8,667 (7.7)		6,597 (10.2)	5,146 (7.9)	
III	17,693 (19.8)	11,171 (10.0)		9,728 (15.0)	9,633 (14.9)	
IV	11,430 (12.8)	9,719 (8.7)		5,875 (9.1)	6,383 (9.8)	
**Chemotherapy**						
No	87,657 (98.2)	111,518 (99.5)	<0.001*	64,073 (98.8)	64,333 (99.2)	<0.001*
Yes	1,581 (1.8)	590 (0.5)		761 (1.2)	501 (0.8)	
**Combined summary stage**						
*In situ*	2,275 (2.5)	2,449 (2.2)	<0.001*	2,258 (3.5)	1,140 (1.8)	<0.001*
Localized	46,247 (51.8)	88,204 (78.7)		42,740 (65.9)	45,127 (69.6)	
Regional	35,993 (40.3)	17,522 (15.6)		17,491 (27.0)	16,151 (24.9)	
Distant	4,723 (5.3)	3,933 (3.5)		2,345 (3.6)	2,416 (3.7)	
**Surgery**						
No	1,472 (1.6)	7,936 (7.1)	<0.001*	1,240 (1.9)	1,384 (2.1)	0.005*
Yes	87,766 (98.4)	104,172 (92.9)		63,594 (98.1)	63,450 (97.9)	
**Income**						
≤ 35,000	1,071 (1.2)	1,384 (1.2)	<0.001*	826 (1.3)	548 (0.8)	<0.001*
35,000–75,000	58,102 (65.1)	71,528 (63.8)		41,995 (64.8)	41,481 (64.0)	
≥75,000	30,065 (33.7)	39,196 (35.0)		22,013 (34.0)	22,805 (35.2)	
Survival months	98.70 ± 63.665	82.72 ± 63.853	<0.001*	99.02 ± 63.941	96.76 ± 67.123	<0.001*
**Survival status**						
Alive	79,316 (88.9)	98,104 (87.5)	<0.001*	57,805 (89.2)	56,914 (87.8)	<0.001*
Other-cause death	8,783 (9.8)	11,951 (10.7)		6,160 (9.5)	6,770 (10.4)	
Cardiac-specific death	1,139 (1.3)	2,053 (1.8)		869 (1.3)	1,150 (1.8)	
**Histologic type ICD-O-3**						
8260–8269	47,168 (52.9)	55,697 (49.7)	<0.001*	31,963 (49.3)	32,516 (50.2)	<0.001*
8340–8349	26,455 (29.6)	36,326 (32.4)		20,834 (32.1)	19,966 (30.8)	
8330–8339	5,865 (6.6)	5,422 (4.8)		4,910 (7.6)	3,266 (5.0)	
8050–8059	4,519 (5.1)	6,384 (5.7)		3,219 (5.0)	4,391 (6.8)	
8290–8299	2,444 (2.7)	2,095 (1.9)		2,081 (3.2)	1,315 (2.0)	
8000–8009	162 (0.2)	1,044 (0.9)		132 (0.2)	286 (0.4)	
8510–8519	395 (0.4)	2,518 (2.2)		242 (0.4)	1,761 (2.7)	
8020–8029	980 (1.1)	809 (0.7)		589 (0.9)	452 (0.7)	
8010–8019	449 (0.5)	1,016 (0.9)		355 (0.5)	394 (0.6)	
8350–8359	351 (0.4)	201 (0.2)		199 (0.3)	159 (0.2)	
Others	450 (0.5)	596 (0.5)		310 (0.5)	328 (0.5)	
**Radiation**						
No	–	112,108	–	–	64,834	
Yes	89,238	–		64,834	–	
Beam radiation	4,225 (4.7)	–		2,473 (4.2)	–	
Combination of beam with implants or isotopes	665 (0.7)	–		407 (0.6)	–	
NOS method or source not specified	524 (0.6)	–		374 (0.6)	–	
Radioactive implants	1,371 (1.5)	–		1,000 (1.5)	–	
Radioisotopes	82,453 (92.4)	–		60,310 (93.0)	–	

*p < 0.05.

After PSM (1:1), 129, 668 patients still retaining, and the distribution of baseline characteristics of the population were similar to before matching. The two groups became statistically indistinguishable in variables of race, origin, and sex, and the differences in other variables also decreased to a certain extent compared to pre-PSM (Table 1).

### Effects of radiation therapy on cardiac-specific death before propensity score matching

**FIGURE 1:**
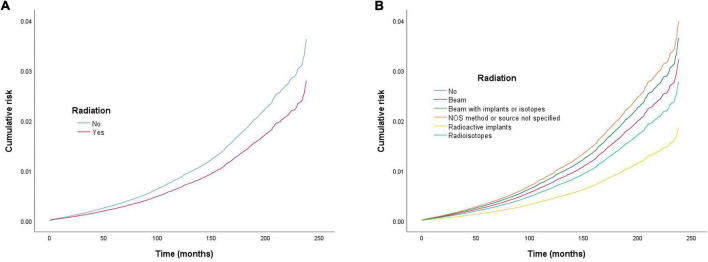
Cumulative risk of cardiac-specific death related to radiation therapy (RT) before propensity score matching (PSM) (the curves of “no radiation” and “beam with implants or radioisotopes” are overlapping and may not be drawn separately). The *Y*-axis of each panel shows the cumulative risk of cardiac-specific death and the *X*-axis shows the time since diagnosis in months. Each line represents the cumulative risk of cardiac-specific death in patients after receiving a treatment. **(A)** Cumulative risk of cardiac-specific death in patients with receiving and not receiving radiotherapy. **(B)** Cumulative risk of cardiacspecific death in patients with not receiving radiotherapy and receiving different modalities of radiotherapy.

**FIGURE 2:**
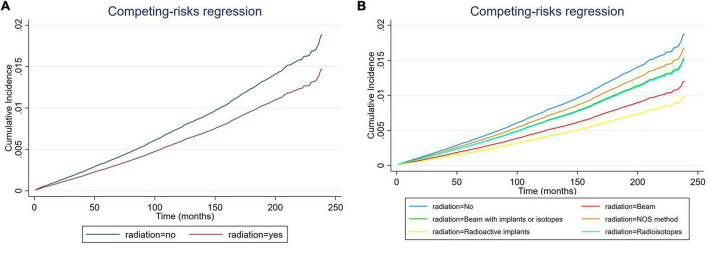
Cumulative incidence rate of cardiac-specific death related to radiation therapy (RT) before propensity score matching (PSM) (the curves of “radioactive isotopes” and “beam with implants or isotopes” are overlapping and may not be drawn separately). The *Y*-axis of each panel shows the cumulative risk of cardiac-specific death and the *X*-axis shows the time since diagnosis in months. Each line represents the cumulative risk of cardiac-specific death in patients after receiving a treatment. **(A)** Cumulative risk of cardiac-specific death in patients with receiving and not receiving radiotherapy. **(B)** Cumulative risk of cardiacspecific death in patients with not receiving radiotherapy and receiving different modalities of radiotherapy.

Multivariate COX regression analysis showed that RT {yes (hazard ratio [HR] = 0.771, 95% confidence interval [CI] = 0.713–0.833, *p* < 0.001) vs. no} was associated with a lower risk of cardiac-specific death among patients with malignant thyroid tumors (Figure 1A). Results of group analysis by type of RT received showed that RT based upon radioisotopes (HR = 0.762, CI = 0.702–0.826, *p* < 0.001) and radioactive implants (HR = 0.51, CI = 0.311–0.835, *p* = 0.007) could reduce the risk of cardiac-specific death with statistical significance. Conversely, RT based upon combination of beam with implants or isotopes (HR = 1.003, CI = 0.636–1.581, *p* = 0.99), as well as RT cases where the type was NOS (not otherwise specified) method (HR = 1.094, CI = 0.604–1.981, *p* = 0.766), could increase the risk of cardiac-specific death – albeit not with statistical significance, while beam radiation (HR = 0.885, CI = 0.706–1.109, *p* = 0.288) therapy alone could reduce the risk of cardiac-specific death, although again not with statistical significance (Figure 1B). The effects of RT on cardiac-specific death were consistent between the Fine-Gray and COX models of regression analysis, with the exception of beam radiation ([HR = 0.638, CI = 0.509–0.800, *p* < 0.001] vs. [HR = 0.885, CI = 0.706–1.109, *p* = 0.288]) alone (Figure 2 and Table 2).

**TABLE 2:** Multivariate regression analyses before propensity score matching.

Variables		COX			Fine-gray	
		Multivariate analysis			Multivariate analysis	
	
	HR	95% CI	*p*	SHR	95% CI	*p*
Age of diagnosis (years)	1.097	1.094–1.101	<0.001*	1.082	1.079–1.085	< 0.001*
**Race**						
**White**						
Black	1.4	1.237–1.585	<0.001*	1.382	1.212–1.575	< 0.001*
Asian or Pacific Islander	0.653	0.562–0.758	<0.001*	0.667	0.572–0.776	< 0.001*
American Indian	0.984	0.602–1.610	0.95	0.971	0.586–1.608	0.908
**Origin**						
Hispanic						
Non-Hispanic	1.19	1.055–1.343	0.005*	1.204	1.063–1.363	0.003*
Sex						
Male						
Female	0.482	0.448–0.520	<0.001*	0.533	0.493–0.575	< 0.001*
**Marital status**						
Single						
Married	0.558	0.503–0.619	<0.001*	0.592	0.532–0.658	< 0.001*
DSW	0.866	0.772–0.971	0.014*	0.873	0.776–0.983	0.025*
Unmarried or domestic partner	1.033	0.385–2.767	0.949	1.368	0.524–3.571	0.522
**Year of diagnosis**						
2000–2004						
2005–2009				0.779	0.712–0.852	< 0.001*
2010–2014				0.627	0.566–0.695	< 0.001*
2015–2019				0.492	0.423–0.572	< 0.001*
Months from diagnosis to treatment	1.007	0.980–1.036	0.598			
**Derived AJCC stage group**						
I						
II	1.111	0.989–1.247	0.075	1.125	1.002–1.264	0.047*
III	1.037	0.913–1.178	0.576	1.032	0.909–1.172	0.624
IV	1.339	1.143–1.569	<0.001*	1.208	1.028–1.420	0.022*
**Chemotherapy**						
No						
Yes	0.698	0.461–1.056	0.089			
**Combined summary stage**						
*In situ*						
Localized	0.707	0.606–0.825	<0.001*	0.773	0.660–0.904	0.001*
Regional	0.764	0.630–0.926	0.006*	0.804	0.663–0.974	0.026*
Distant	0.82	0.643–1.047	0.111	0.600	0.467–0.772	< 0.001*
**Surgery**						
No						
Yes	0.462	0.407–0.523	<0.001*	0.864	0.748–0.998	0.046*
**Income**						
≤35,000						
35,000–75,000	0.864	0.65–1.15	0.317	0.915	0.681–1.228	0.552
≥75,000	0.662	0.496–0.885	0.005*	0.729	0.540–0.983	0.038*
**Histologic type**						
8260–8269						
8340–8349	1.072	0.984–1.169	0.111	1.079	0.989–1.178	0.088
8330–8339	1.159	1.008–1.331	0.038*	1.066	0.924–1.229	0.379
8050–8059	1.215	1.072–1.377	0.002*	1.215	1.068–1.383	0.003*
8290–8299	1.164	0.983–1.378	0.078	1.116	0.942–1.323	0.206
8000–8009	0.692	0.531–0.903	0.007*	0.627	0.462–0.851	0.003*
8510–8519	1.164	0.924–1.468	0.198	1.021	0.804–1.296	0.864
8020–8029	1.398	0.919–2.128	0.118	0.268	0.163–0.440	< 0.001*
8010–8019	0.784	0.59–1.04	0.092	0.660	0.482–0.904	0.010*
8350–8359	1.323	0.66–2.653	0.431	1.111	0.536–2.307	0.777
Others	1.317	0.895–1.937	0.163	0.676	0.445–1.028	0.067
**Radiation**						
No						
Yes	0.771	0.713–0.833	<0.001*	0.778	0.720–0.841	< 0.001*
Beam radiation	0.885	0.706–1.109	0.288	0.638	0.509–0.800	< 0.001*
Combination of beam with implants or isotopes	1.003	0.636–1.581	0.99	0.811	0.511–1.286	0.373
NOS method or source not specified	1.094	0.604–1.981	0.766	0.893	0.483–1.649	0.717
Radioactive implants	0.51	0.311–0.835	0.007*	0.523	0.322–0.849	0.009*
Radioisotopes	0.762	0.702–0.826	<0.001*	0.799	0.737–0.867	< 0.001*

*p < 0.05.

### Other influencing factors for cardiac-specific death before propensity score matching

Multivariate COX model regression analysis showed that race (Asian or Pacific Islander ethnicity [HR = 0.653, CI = 0.562–0.758, *p* < 0.001] vs. White ethnicity), sex (female [HR = 0.482, CI = 0.448–0.520, *p* < 0.001] vs. male), marital status (married [HR = 0.558, CI = 0.503–0.619, *p* < 0.001], DSW (divorced, separated, and widowed) [HR = 0.866, CI = 0.772–0.971, *p* = 0.014] vs. single), combined summary stage (localized [HR = 0.707, CI = 0.606–0.825, *p* < 0.001], regional [HR = 0.764, CI = 0.630–0.926, *p* = 0.006] vs. *in situ*), surgery (yes [HR = 0.462, CI = 0.407–0.523, *p* < 0.001] vs. no), income (≥75,000 [HR = 0.662, CI = 0.496–0.885, *p* = 0.005] vs. ≤35,000), and histologic type (8000–8009 [HR = 0.692, CI = 0.5316–0.903, *p* = 0.007] vs. 8260–8269) were associated with lower risk of cardiac-specific death, while older age of diagnosis (HR = 1.097, CI = 1.094–1.101, *p* < 0.001), race (African ethnicity [HR = 1.4, CI = 1.237–1.585, *p* < 0.001] vs. White ethnicity), Origin (non-Hispanic ancestry [HR = 1.19, CI = 1.055–1.343, *p* = 0.005] vs. Hispanic ancestry), derived AJCC stage (IV [HR = 1.339, CI = 1.143–1.569, *p* < 0.001] vs. I), and histologic type (8330–8339 [HR = 1.159, CI = 1.008–1.331, *p* = 0.038] and 8050–8059 [HR = 1.215, CI = 1.072–1.377, *p* = 0.002] vs. 8260–8269) were risk factors for cardiac-specific death.

All variables tested for their impact on cardiac-specific death–with the exception of the year of diagnosis, derived AJCC stage group (II), combined summary stage (distant), histologic type (8330–8339, 8020–8029, 8010–8019)–were in agreement between the Fine-Gray and COX models of regression analysis (Table 2).

### Effects of radiation therapy on cardiac-specific death after propensity score matching

**FIGURE 3:**
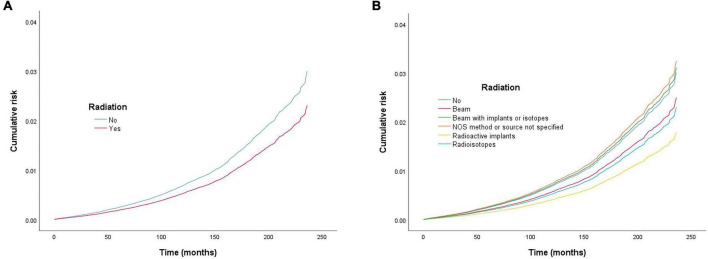
Cumulative risk of cardiac-specific death related to radiation therapy (RT) after propensity score matching (PSM). The *Y*-axis of each panel shows the cumulative risk of cardiac-specific death and the *X*-axis shows the time since diagnosis in months. Each line represents the cumulative risk of cardiac-specific death in patients after receiving a treatment. **(A)** Cumulative risk of cardiac-specific death in patients with receiving and not receiving radiotherapy. **(B)** Cumulative risk of cardiacspecific death in patients with not receiving radiotherapy and receiving different modalities of radiotherapy.

**FIGURE 4:**
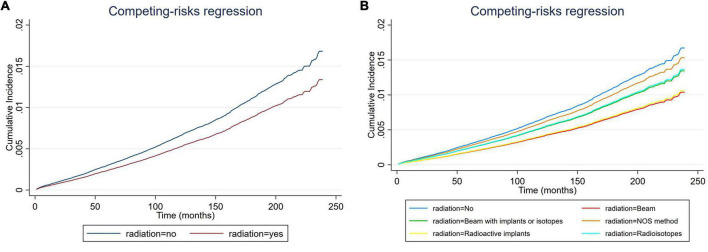
Cumulative incidence rate of cardiac-specific death related to radiation therapy (RT) after propensity score matching (PSM) (the curves of “radioactive isotopes” and “beam with implants or isotopes” are overlapping and may not be drawn separately, as was the curves of “beam” and “radioactive implants”). The *Y*-axis of each panel shows the cumulative risk of cardiac-specific death and the *X*-axis shows the time since diagnosis in months. Each line represents the cumulative risk of cardiac-specific death in patients after receiving a treatment. **(A)** Cumulative risk of cardiac-specific death in patients with receiving and not receiving radiotherapy. **(B)** Cumulative risk of cardiacspecific death in patients with not receiving radiotherapy and receiving different modalities of radiotherapy.

Multivariate COX model regression analysis showed that RT {yes (HR = 0.770, 95%, CI = 0.704–0.843, *p* < 0.001) vs. no} was associated with a lower risk of cardiac-specific death among patients with malignant thyroid tumors (Figure 3A). Results of group analysis by the types of RT received showed that RT based upon radioisotopes (HR = 0.764, CI = 0.696–0.839, *p* < 0.001) and radioactive implants (HR = 0.596, CI = 0.357–0.992, *p* = 0.047) could reduce the risk of cardiac-specific death with statistical significance. Conversely, RT based upon combination of beam with implants or isotopes (HR = 1.033, CI = 0.597–1.788, *p* = 0.908) therapy, as well as RT cases where the type was NOS (not otherwise specified) method (HR = 1.079, CI = 0.559–2.083, *p* = 0.821), could increase the risk of cardiac-specific death – albeit not with statistical significance, while beam radiation (HR = 0.829, CI = 0.631–1.089, *p* = 0.179) therapy alone could reduce the risk of cardiac-specific death, although again not with statistical significance (Figure 3B). The effects of RT on cardiac-specific death were consistent between the Fine-Gray and COX model of regression analysis, with the exception of beam radiation ([HR = 0.619, CI = 0.471–0.815, *p* = 0.001] vs. [HR = 0.829, CI = 0.631–1.089, *p* = 0.179]) and radioactive implants ([HR = 0.634, CI = 0.385–1.044, *p* = 0.073] vs. [HR = 0.596, CI = 0.357–0.992, *p* = 0.047]; Figure 4 and Table 3).

**TABLE 3:** Multivariate regression analyses after propensity score matching.

Variables		COX			Fine-gray	
		Multivariate analysis			Multivariate analysis	
	
	HR	95% CI	*p*	SHR	95% CI	*p*
Age of diagnosis (years)	1.101	1.096–1.105	<0.001*	1.082	1.079–1.086	< 0.001*
**Race**						
**White**						
Black	1.351	1.145–1.593	<0.001*	1.346	1.136–1.594	0.001*
Asian or Pacific Islander	0.654	0.545–0.785	<0.001*	0.673	0.561–0.809	< 0.001*
American Indian	0.756	0.392–1.460	0.405	0.826	0.425–1.605	0.573
**Origin**						
**Hispanic**						
Non-Hispanic	1.228	1.059–1.425	0.007*	1.235	1.062–1.436	0.006*
**Sex**						
Male						
Female	0.462	0.421–0.507	<0.001*	0.507	0.461–0.558	< 0.001*
**Marital status**						
Single						
Married	0.533	0.468–0.607	<0.001*	0.574	0.503–0.654	< 0.001*
DSW	0.886	0.767–1.023	0.1	0.889	0.768–1.029	0.116
Unmarried or domestic partner	1.507	0.482–4.710	0.48	1.981	0.673–5.834	0.215
**Year of diagnosis**						
2000–2004						
2005–2009				0.770	0.691–0.858	< 0.001*
2010–2014				0.626	0.548–0.715	< 0.001*
2015–2019				0.490	0.397–0.604	< 0.001*
Months from diagnosis to treatment	1.006	0.973–1.040	0.726	1.008	0.975–1.042	0.630
**Derived AJCC stage group**						
I						
II	1.141	0.993–1.311	0.063	1.149	0.999–1.322	0.052
III	1.136	0.978–1.320	0.096	1.121	0.967–1.299	0.131
IV	1.673	1.369–2.045	<0.001*	1.435	1.175–1.752	< 0.001*
**Chemotherapy**						
No						
Yes	0.650	0.384–1.101	0.109			
**Combined summary stage**						
*In situ*						
Localized	0.697	0.575–0.846	<0.001*	0.773	0.635–0.940	0.010*
Regional	0.689	0.542–0.877	0.002*	0.745	0.587–0.946	0.016*
Distant	0.69	0.501–0.951	0.023*	0.534	0.386–0.740	< 0.001*
**Surgery**						
No						
Yes	0.508	0.401–0.645	<0.001*	1.006	0.777–1.302	0.966
**Income**						
≤35,000						
35,000–75,000	0.75	0.507–1.108	0.149	0.869	0.580–1.303	0.497
≥75,000	0.592	0.398–0.880	0.01*	0.696	0.462–1.049	0.083
**Histologic type**						
8260–8269						
8340–8349	1.049	0.942–1.169	0.382	1.066	0.956–1.189	0.250
8330–8339	1.098	0.924–1.305	0.286	1.004	0.842–1.198	0.961
8050–8059	1.259	1.08–1.467	0.003*	1.289	1.103–1.507	0.001*
8290–8299	1.181	0.97–1.438	0.097	1.114	0.913–1.360	0.287
8000–8009	0.914	0.587–1.422	0.689	0.725	0.444–1.183	0.198
8510–8519	1.185	0.891–1.576	0.243	1.029	0.764–1.386	0.850
8020–8029	1.711	0.995–2.943	0.052	0.297	0.163–0.540	< 0.001*
8010–8019	1.323	0.9–1.944	0.155	0.883	0.575–1.357	0.570
8350–8359	0.867	0.324–2.317	0.776	0.843	0.325–2.184	0.725
Others	1.358	0.823–2.242	0.231	0.724	0.427–1.227	0.231
**Radiation**						
No						
Yes	0.770	0.704–0.843	<0.001*	0.794	0.725–0.869	< 0.001*
Beam radiation	0.829	0.631–1.089	0.179	0.619	0.471–0.815	0.001*
Combination of beam with implants or isotopes	1.033	0.597–1.788	0.908	0.803	0.458–1.408	0.444
NOS method or source not specified	1.079	0.559–2.083	0.821	0.916	0.461–1.819	0.802
Radioactive implants	0.596	0.357–0.992	0.047*	0.634	0.385–1.044	0.073
Radioisotopes	0.764	0.696–0.839	<0.001*	0.816	0.742–0.897	< 0.001*

*p < 0.05.

### Other influencing factors for cardiac-specific death after propensity score matching

Multivariate COX model regression analysis showed that race (Asian or Pacific Islander ethnicity [HR = 0.654, CI = 0.545–0.785, *p* < 0.001] vs. White ethnicity), sex (female [HR = 0.462, CI = 0.421–0.507, *p* < 0.001] vs. male), marital status (married [HR = 0.533, CI = 0.468–0.607, *p* < 0.001] vs. single), combined summary stage (localized [HR = 0.697, CI = 0.575–0.846, *p* < 0.001], regional [HR = 0.689, CI = 0.542–0.877, *p* = 0.002], distant [HR = 0.690, CI = 0.501–0.951, *p* = 0.023] vs. *in situ*), surgery (yes [HR = 0.508, CI = 0.401–0.645, *p* < 0.001] vs. no), and income (≥75,000 [HR = 0.592, CI = 0.398–0.880, *p* = 0.01] vs. ≤35,000) were associated with lower risk of cardiac-specific death, while older age of diagnosis (HR = 1.101, CI = 1.096–1.105, *p* < 0.001), race (African ethnicity [HR = 1.351, CI = 1.145–1.593, *p* < 0.001] vs. White ethnicity), Origin (non-Hispanic ancestry [HR = 1.228, CI = 1.059–1.425, *p* = 0.007] vs. Hispanic ancestry), derived AJCC stage (IV [HR = 1.673, CI = 1.369–2.045, *p* < 0.001] vs. I), and histologic type (8050–8059 [HR = 1.259, CI = 1.08–1.467, *p* = 0.003] vs. 8260–8269) were risk factors for cardiac-specific death.

All variables tested for their impact on cardiac-specific death – with the exception of the year of diagnosis, surgery, income, histologic type (8020–8029) – were in agreement between the Fine-Gray and COX models of regression analysis (Table 3).

## Discussion

Previous studies have reported that patients with thyroid cancer have no increased risk of dying from cardiovascular disease relative to the general population, but differences have been shown depending on the year, and the highest rates of heart disease-specific survival across various cancer types is observed in patients with thyroid cancer (11–13). Of course, there are also studies with opposite results showing a significant increase in cardiovascular as well as all-cause mortality in patients with thyroid cancer (14). These discrepancies may in part be due to variable patient baseline characteristics as some studies did not fully consider the effect of use or type of RT. Additionally, histological variations can also lead to different survival outcomes as the results of our study showed. Although previous studies have shown that patients with thyroid cancer may not have an increased risk of cardiovascular death as same as other cancers, the exact cause of this has not been clarified. Our study unexpectedly found that RT based upon beam radiation, radioisotopes and radioactive implant may be associated with lower risk of cardiac-specific death in patients with malignant thyroid tumors. Considering that 43.7% of patients received above three types of RT, we speculate that perhaps the low risk of cardiac-specific death in patients with thyroid cancer was associated with RT. Additionally, before PSM, the incidence of cardiac-specific death was lower in the RT group than in the non-RT group (1.2 vs. 1.8%, *p* < 0.001), with the incidence of cardiac death being lower in the groups of beam radiation (2.1%) and combination of beam with implants or isotopes (2.8%), NOS method or source not specified (2.0%) had higher incidences of cardiac death than the non-RT group, and the incidences of cardiac-specific death in groups receiving radioactive implants (1.1%) and radioisotopes (1.2%) were lower than that in the non-RT group (1.8%), but only the group receiving radioisotopes therapy reached statistical difference. The situation after PSM was similar to that before PSM, except for groups receiving NOS method or source not specified or combination of beam with implants or isotopes, RT based upon beam radiation, radioactive implants and radioisotopes reduced the risk of cardiac-specific death compared to non-RT. These results reflect the role of RT in decreasing the cardiac-specific deaths of patients with malignant thyroid tumors in a side-by-side manner, contradicting previous concept that RT generally increases cardiac-specific death. We know that everything has two sides, the overall effect is reflected in the offset of the positive and negative effects, and RT is no exception. RT can lead to direct toxic and negative effects on the heart, of course, it can also bring positive effects on the heart. The effect of radiation on the heart may be caused in a variety of ways, of course, the exact mechanism still needs further research. In addition, our study found some differences in the effects of different modalities of RT on the heart, after adjusting for multivariate confounding variables, i.e., RT based upon radioisotopes and radioactive implants were associated with lower risk of cardiac-specific death, beam radiation may had a similar effect, and the remaining RT methods did not significantly increase the risk of cardiac-specific death after comprehensive consideration. We think the result of radioisotopes is more credible as far more people (92.4%) receive this form of treatment than any others. In addition to effective treatment, internal RT can produce a better killing effect on cancer cells while minimizing non-targeted irradiation to surrounding healthy tissue (15).

Cancer patients have a consistently higher risk of death due to cardiovascular diseases compared to the general United States population, and this risk is inversely related to the age at diagnosis (11). Our study found that this trend also applies to patients with thyroid cancer. Previous studies have also shown that cardiovascular mortality is usually higher among patients with African ancestry, and lower among patients with Caucasian, Asian and Hispanic ancestry (16). Consistent with this, we found that Caucasian, Asian or Pacific Islander, and Hispanic ancestry were protective factors in reducing post-radiation cardiac-specific death in patients with malignant thyroid tumors, while African ancestry was a risk factor. Previous studies have shown that women have lower risk factors for most causes of death (17), a self-reported “good marital status” is a protective factor for reducing cardiovascular events, and being single, experiencing marital stress, or experiencing a divorce all increase risk of cardiovascular death (18, 19). Our results also showed that female sex and marital status were protective factors of radiation-induced cardiac-specific death in our cohort. Lower socioeconomic status may also continue to contribute to increased cancer rates and increased risk of death from cardiovascular disease in cancer survivors (20). Our study showed that higher income could reduce the risk of cardiac-specific death in patients with malignant thyroid tumors, but without statistical significance after PSM. It is possible that this may be due to bias before the PSM, as PSM could correct for some deviations in baseline characteristics of the two groups. In addition, hypofractionated or dedifferentiated cancer (histological type 8020–8029) was associated with a lower risk of cardiac-specific death in our study. Except for higher age (average age about 50 years old), patients included were predominantly Caucasian, female sex, non-Hispanic ancestry, marital status, Derived AJCC Stage Group I, non-chemotherapy, and moderate to high income, all of which were associated with a lower risk of cardiovascular mortality. Thus, the population included in this study was considered to have a lower risk of cardiovascular mortality, and the influence of confounding factors on the effect of RT action was small.

The primary differences between the results of the COX and Fine-Gray model analyses were that the statistical difference between the effect of surgery and radioactive implant treatments on cardiac-specific death were lost, Additionally, later year of diagnosis and receipt of beam radiation therapy became significant protective factors in reducing cardiac-specific death in the Fine-Gray model analysis, unlike in the COX model analysis. This may be caused by the different effects of the models, as the Fine-Gray model has the advantages of analysis and the results are more reliable in the presence of competitive death.

## Limitation

Although this is the first study analyzing the effect of different types of RT on the risks of cardiac-specific death in patients with malignant thyroid tumors, it has a very severe limitation that ought to be considered. The SEER database is not allowing to evaluate any kind of clinical characteristics, like cardiovascular risk factors, type of cardiac deaths, comorbidities and so on. This limitation prevent us from analysing the results according to cardiovascular risk factors and clarifying the causes of death. Hence, our results only suggest (not demonstrating) that RT may not be dangerous or may be protective for the heart.

## Conclusion

This study showed the factors influencing cardiac-specific death in patients with thyroid malignancies, and found a phenomenon whereby radioisotopes and radioactive implant therapies were associated with reduced risks of cardiac-specific death in patients with thyroid malignancy. Thus, our results may suggest additional effects of radiation on the heart that differ across different types of RT. These results lay the foundation for identification of the risk factors of cardiac-specific death among patients with thyroid malignancies after undergoing RT, and may allowing improvement of the clinical application of RT in thyroid malignancies without as much concern regarding adverse cardiac effects. Importantly, however, these findings should be confirmed by special randomized controlled trials.

## Data availability statement

The raw data supporting the conclusions of this article will be made available by the authors, without undue reservation.

## Ethics statement

Ethical review and approval and patient consent were not required for this study in accordance with the local legislation and institutional requirements.

## Author contributions

RW and HY contributed to the conception, design of the study, analyzed, and interpreted the data. YW and JW did the literature search and applied the inclusion and exclusion criteria. RW and LM contributed to the collection and assembly of data. RW, LW, YW, and XZ contributed to the writing of the report. All authors approved the final version of the report.
