# Supplementary material for: Effects of different types of radiation therapy on cardiac-specific death in patients with thyroid malignancy
Source: Front Cardiovasc Med. 2022 Nov 10;9:996732. doi: 10.3389/fcvm.2022.996732 (PMC9691335; doi:10.3389/fcvm.2022.996732)
Supplement: Supplementary file 1 [file Data_Sheet_1.docx]

**Table 1** Univariate regression analyses before propensity score matching.

| Variables |  | COX |  |  | Fine-Gray |  |
| --- | --- | --- | --- | --- | --- | --- |
|  |  | Univariate analysis |  |  | Univariate analysis |  |
|  | HR | 95% CI | P | SHR | 95% CI | P |
| Age of diagnosis (years) | 1.106 | 1.103-1.109 | <0.001 | 1.083 | 1.080-1.086 | <0.001 |
| Race |  |  |  |  |  |  |
| White |  |  |  |  |  |  |
| Black | 1.453 | 1.288-1.640 | <0.001 | 1.371 | 1.210-1.553 | <0.001 |
| Asian or Pacific Islander | 0.569 | 0.491-0.659 | <0.001 | 0.563 | 0.485-0.655 | <0.001 |
| American Indian | 0.774 | 0.473-1.265 | 0.306 | 0.778 | 0.477-1.269 | 0.315 |
| Origin |  |  |  |  |  |  |
| Hispanic |  |  |  |  |  |  |
| Non-Hispanic | 1.633 | 1.450-1.838 | <0.001 | 1.647 | 1.458-1.861 | <0.001 |
| Sex |  |  |  |  |  |  |
| Male |  |  |  |  |  |  |
| Female | 0.406 | 0.378-0.436 | <0.001 | 0.447 | 0.416-0.480 | <0.001 |
| Marital status |  |  |  |  |  |  |
| Single |  |  |  |  |  |  |
| Married | 1.129 | 1.020-1.249 | 0.019 | 1.121 | 1.011-1.243 | 0.031 |
| DSW | 3.512 | 3.150-3.914 | <0.001 | 3.143 | 2.814-3.510 | <0.001 |
| Unmarried or Domestic Partner | 1.708 | 0.638-4.573 | 0.286 | 1.749 | 0.658-4.453 | 0.263 |
| Year of diagnosis |  |  |  |  |  |  |
| 2000-2004 |  |  |  |  |  |  |
| 2005-2009 | 0.987 | 0.901-1.081 | 0.774 | 0.923 | 0.849-1.014 | 0.097 |
| 2010-2014 | 0.973 | 0.875-1.082 | 0.611 | 0.879 | 0.793-0.975 | 0.015 |
| 2015-2019 | 0.982 | 0.844-1.142 | 0.814 | 0.817 | 0.701-0.951 | 0.009 |
| Months from diagnosis to treatment | 1.031 | 1.005-1.057 | 0.020 | 1.026 | 1.000-1.053 | 0.053 |
| Derived AJCC Stage Group |  |  |  |  |  |  |
| Ⅰ |  |  |  |  |  |  |
| Ⅱ | 1.904 | 1.701-2.131 | <0.001 | 1.834 | 1.636-2.055 | <0.001 |
| Ⅲ | 1.691 | 1.532-1.867 | <0.001 | 1.624 | 1.470-1.794 | <0.001 |
| Ⅳ | 3.560 | 3.241-3.910 | <0.001 | 2.426 | 2.202-2.674 | <0.001 |
| Chemotherapy |  |  |  |  |  |  |
| No |  |  |  |  |  |  |
| Yes | 1.881 | 1.278-2.768 | 0.001 | 0.731 | 0.489-1.093 | 0.126 |
| Combined Summary Stage |  |  |  |  |  |  |
| In situ |  |  |  |  |  |  |
| Localized | 0.805 | 0.693-0.935 | 0.005 | 0.769 | 0.662-0.894 | 0.001 |
| Regional | 0.780 | 0.664-0.917 | 0.003 | 0.730 | 0.621-0.858 | <0.001 |
| Distant | 2.611 | 2.162-3.153 | <0.001 | 1.393 | 1.149-1.689 | 0.001 |
| Surgery |  |  |  |  |  |  |
| No |  |  |  |  |  |  |
| Yes | 0.176 | 0.159-0.196 | <0.001 | 0.349 | 0.311-0.392 | <0.001 |
| Income |  |  |  |  |  |  |
| ≤35,000 |  |  |  |  |  |  |
| 35,000-75,000 | 0.731 | 0.551-0.970 | 0.030 | 0.764 | 0.573-1.020 | 0.067 |
| ≥75,000 | 0.527 | 0.395-0.702 | <0.001 | 0.562 | 0.420-0.754 | <0.001 |
| Histologic Type |  |  |  |  |  |  |
| 8260-8269 |  |  |  |  |  |  |
| 8340-8349 | 1.414 | 1.049-1.241 | 0.002 | 1.170 | 1.075-1.273 | <0.001 |
| 8330-8339 | 1.629 | 1.423-1.866 | <0.001 | 1.579 | 1.376-1.812 | <0.001 |
| 8050-8059 | 1.286 | 1.136-1.457 | <0.001 | 1.339 | 1.181-1.519 | <0.001 |
| 8290-8299 | 2.335 | 1.977-2.758 | <0.001 | 2.270 | 1.920-2.683 | <0.001 |
| 8000-8009 | 5.393 | 4.170-6.974 | <0.001 | 3.217 | 2.414-4.286 | <0.001 |
| 8510-8519 | 2.191 | 1.742-2.754 | <0.001 | 1.866 | 1.475-2.361 | <0.001 |
| 8020-8029 | 7.614 | 5.149-11.259 | <0.001 | 0.921 | 0.569-1.489 | 0.736 |
| 8010-8019 | 3.951 | 3.000-5.202 | <0.001 | 2.467 | 1.835-3.319 | <0.001 |
| 8350-8359 | 1.007 | 0.502-2.018 | 0.984 | 0.896 | 0.428-1.872 | 0.770 |
| others | 3.077 | 2.101-4.506 | <0.001 | 1.855 | 1.241-2.772 | 0.003 |
| Radiation |  |  |  |  |  |  |
| No |  |  |  |  |  |  |
| Yes | 0.578 | 0.538-0.621 | <0.001 | 0.617 | 0.573-0.663 | <0.001 |
| Beam radiation | 1.695 | 1.374-2.092 | <0.001 | 1.020 | 0.823-1.264 | 0.857 |
| Combination of beam with implants or isotopes | 1.391 | 0.885-2.185 | 0.152 | 1.253 | 0.797-1.970 | 0.329 |
| NOS method or source not specified | 0.905 | 0.500-1.637 | 0.741 | 0.918 | 0.509-1.656 | 0.776 |
| Radioactive implants | 0.408 | 0.249-0.667 | <0.001 | 0.446 | 0.273-0.729 | 0.001 |
| Radioisotopes | 0.541 | 0.502-0.583 | <0.001 | 0.592 | 0.549-0.638 | <0.001 |

**Table 2** Univariate regression analyses after propensity score matching.

| Variables |  | COX |  |  | Fine-Gray |  |
| --- | --- | --- | --- | --- | --- | --- |
|  |  | Univariate analysis |  |  | Univariate analysis |  |
|  | HR | 95% CI | P | SHR | 95% CI | P |
| Age of diagnosis (years) | 1.109 | 1.105-1.113 | <0.001 | 1.084 | 1.081-1.087 | <0.001 |
| Race |  |  |  |  |  |  |
| White |  |  |  |  |  |  |
| Black | 1.317 | 1.121-1.547 | 0.001 | 1.278 | 1.086-1.505 | 0.003 |
| Asian or Pacific Islander | 0.590 | 0.493-0.706 | <0.001 | 0.590 | 0.492-0.707 | <0.001 |
| American Indian | 0.677 | 0.351-1.302 | 0.242 | 0.665 | 0.346-1.276 | 0.220 |
| Origin |  |  |  |  |  |  |
| Hispanic |  |  |  |  |  |  |
| Non-Hispanic | 1.601 | 1.383-1.853 | <0.001 | 1.612 | 1.390-1.869 | <0.001 |
| Sex |  |  |  |  |  |  |
| Male |  |  |  |  |  |  |
| Female | 0.368 | 0.337-0.402 | <0.001 | 0.405 | 0.371-0.443 | <0.001 |
| Marital status |  |  |  |  |  |  |
| Single |  |  |  |  |  |  |
| Married | 1.125 | 0.991-1.277 | 0.069 | 1.106 | 0.974-1.256 | 0.121 |
| DSW | 3.55 | 3.097-4.069 | <0.001 | 3.130 | 2.728-3.590 | <0.001 |
| Unmarried or Domestic Partner | 2.32 | 0.744-7.239 | 0.147 | 2.209 | 0.716-6.821 | 0.168 |
| Year of diagnosis |  |  |  |  |  |  |
| 2000-2004 |  |  |  |  |  |  |
| 2005-2009 | 0.944 | 0.845-1.053 | 0.300 | 0.885 | 0.796-0.985 | 0.025 |
| 2010-2014 | 0.917 | 0.801-1.051 | 0.215 | 0.831 | 0.728-0.948 | 0.006 |
| 2015-2019 | 0.861 | 0.694-1.069 | 0.175 | 0.760 | 0.615-0.940 | 0.011 |
| Months from diagnosis to treatment | 1.048 | 1.018-1.079 | 0.002 | 1.041 | 1.012-1.070 | 0.005 |
| Derived AJCC Stage Group |  |  |  |  |  |  |
| Ⅰ |  |  |  |  |  |  |
| Ⅱ | 2.083 | 1.819-2.385 | <0.001 | 1.985 | 1.733-2.273 | <0.001 |
| Ⅲ | 2.235 | 1.985-2.515 | <0.001 | 2.064 | 1.833-2.324 | <0.001 |
| Ⅳ | 4.346 | 3.846-4.911 | <0.001 | 2.908 | 2.569-3.293 | <0.001 |
| Chemotherapy |  |  |  |  |  |  |
| No |  |  |  |  |  |  |
| Yes | 2.247 | 1.373-3.677 | 0.001 | 0.818 | 0.491-1.362 | 0.440 |
| Combined Summary Stage |  |  |  |  |  |  |
| In situ |  |  |  |  |  |  |
| Localized | 0.848 | 0.702-1.023 | 0.085 | 0.823 | 0.682-0.994 | 0.043 |
| Regional | 1.047 | 0.856-1.282 | 0.653 | 0.966 | 0.790-1.181 | 0.737 |
| Distant | 3.367 | 2.642-4.291 | <0.001 | 1.753 | 1.372-2.241 | <0.001 |
| Surgery |  |  |  |  |  |  |
| No |  |  |  |  |  |  |
| Yes | 0.151 | 0.124-0.184 | <0.001 | 0.402 | 0.324-0.500 | <0.001 |
| Income |  |  |  |  |  |  |
| ≤35,000 |  |  |  |  |  |  |
| 35,000-75,000 | 0.743 | 0.504-1.095 | 0.133 | 0.800 | 0.539-1.188 | 0.269 |
| ≥75,000 | 0.543 | 0.366-0.804 | 0.002 | 0.597 | 0.400-0.891 | 0.012 |
| Histologic Type |  |  |  |  |  |  |
| 8260-8269 |  |  |  |  |  |  |
| 8340-8349 | 1.157 | 1.04-1.287 | 0.007 | 1.177 | 1.058-1.310 | 0.003 |
| 8330-8339 | 1.573 | 1.331-1.860 | <0.001 | 1.519 | 1.283-1.798 | <0.001 |
| 8050-8059 | 1.318 | 1.132-1.535 | <0.001 | 1.385 | 1.190-1.613 | <0.001 |
| 8290-8299 | 2.549 | 2.101-3.092 | <0.001 | 2.420 | 1.993-2.939 | <0.001 |
| 8000-8009 | 4.827 | 3.128-7.449 | <0.001 | 3.199 | 2.000-5.114 | <0.001 |
| 8510-8519 | 2.254 | 1.701-2.986 | <0.001 | 1.827 | 1.363-2.449 | <0.001 |
| 8020-8029 | 10.065 | 6.111-16.576 | <0.001 | 1.107 | 0.624-1.963 | 0.729 |
| 8010-8019 | 3.916 | 2.684-5.712 | <0.001 | 2.573 | 1.722-3.844 | <0.001 |
| 8350-8359 | 0.84 | 0.314-2.244 | 0.728 | 0.827 | 0.312-2.191 | 0.703 |
| others | 2.923 | 1.781-4.797 | <0.001 | 1.928 | 1.167-3.186 | 0.010 |
| Radiation |  |  |  |  |  |  |
| No |  |  |  |  |  |  |
| Yes | 0.746 | 0.683-0.815 | <0.001 | 0.768 | 0.702-0.839 | <0.001 |
| Beam radiation | 2.224 | 1.732-2.856 | <0.001 | 1.277 | 0.989-1.648 | 0.060 |
| Combination of beam with implants or isotopes | 1.746 | 1.01-3.016 | 0.046 | 1.529 | 0.886-2.640 | 0.127 |
| NOS method or source not specified | 1.259 | 0.653-2.426 | 0.492 | 1.230 | 0.641-2.361 | 0.533 |
| Radioactive implants | 0.647 | 0.389-1.078 | 0.094 | 0.683 | 0.410-1.137 | 0.143 |
| Radioisotopes | 0.699 | 0.638-0.766 | <0.001 | 0.736 | 0.671-0.807 | <0.001 |
